# Supplementary material for: The causal relationship between the human gut microbiota and pyogenic arthritis: a Mendelian randomization study
Source: Front Cell Infect Microbiol. 2024 Nov 26;14:1452480. doi: 10.3389/fcimb.2024.1452480 (PMC11629706; doi:10.3389/fcimb.2024.1452480)
Supplement: Supplementary file 1 [file DataSheet1.zip › mendelian test/ebi-a-GCST90027807.csv_leave_one_out.pdf]

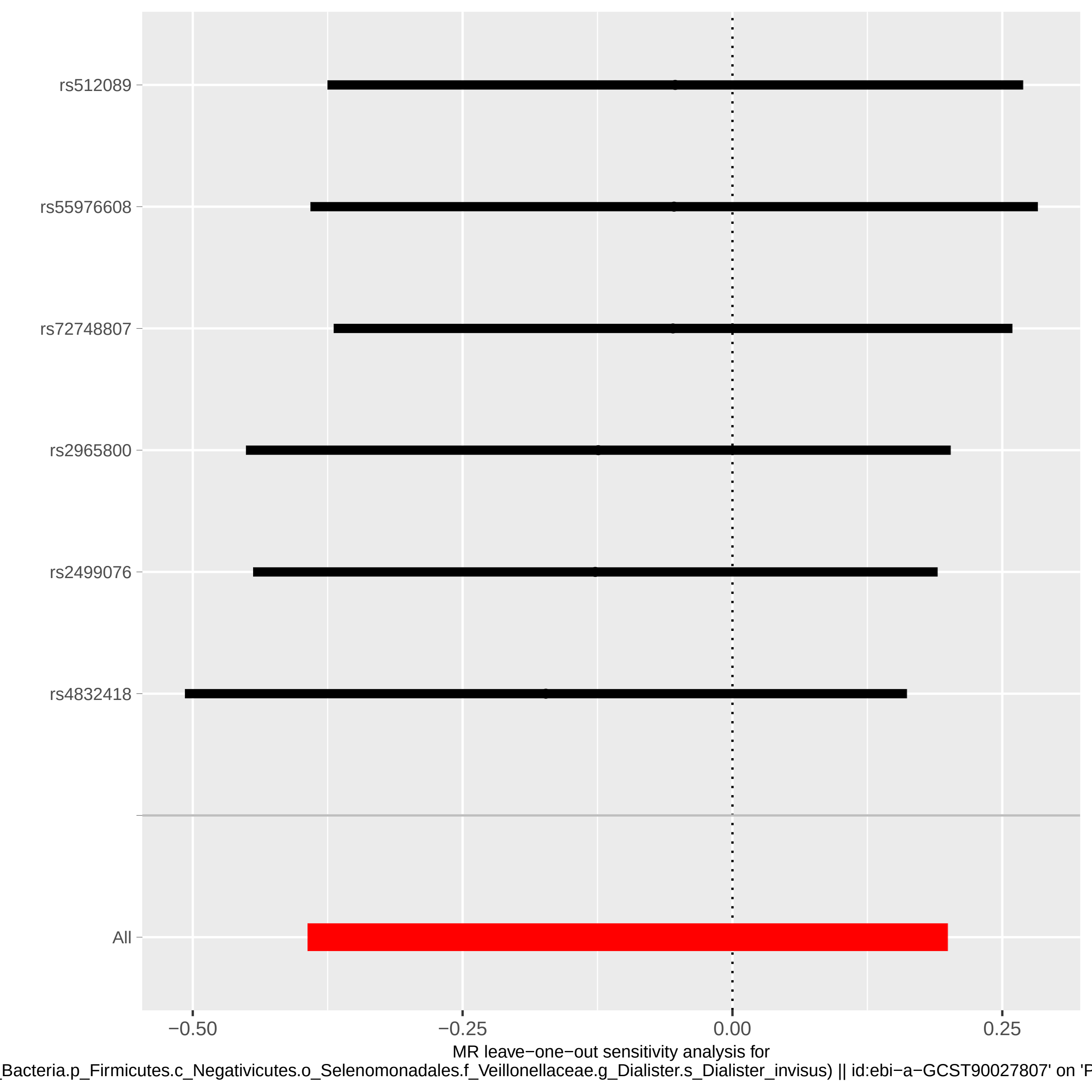
rs512089
rs55976608
rs72748807
rs2965800
rs2499076
rs4832418
All
−0.50
−0.25
0.00
0.25
MR leave−one−out sensitivity analysis for
_Bacteria.p_Firmicutes.c_Negativicutes.o_Selenomonadales.f_Veillonellaceae.g_Dialister.s_Dialister_invisus) || id:ebi−a−GCST90027807' on 'P
